# Prostate Cancer: Current Treatment and Prevention Strategies

**DOI:** 10.5812/ircmj.6499

**Published:** 2013-04-05

**Authors:** Fang-zhi Chen, Xiao-kun Zhao

**Affiliations:** 1 Department of Urology, the Second Xiangya Hospital of Central South University, Changsha, Hunan, China

**Keywords:** Prostate Cancer, Radiation Oncology, Radiotherapy, Proton Therapy, Maintenance Chemotherapy, Cryosurgery, Diet Therapy

## Abstract

**Abstract:**

Prostate cancer is one of the life threatening disorders of male. Although, over the last two decades, a high rate of overdiagnosis, and overtreatment has lowered the incidence rate of prostate cancer, the treatment or prevention strategies are not enough to control the high rate of disease related mortality. Current medical treatment approaches include surgery, radiation therapy, chemotherapy, hormonal therapy, cryosurgery and other methods. These approaches are more or less effective either as monotherapy or in multimodal approach. However, many adverse or side effects exist with these strategies. Researches are ongoing to find out the way or better strategies to eliminate the adverse effects. Dietary modifications may also contribute to decrease prostate cancer risk. Several nutraceuticals against prostate cancer have also been identified. This review article summarizes some of the current treatment, and prevention strategies with the protection of prostate cancer, which may be helpful to control and prevent this highly frequent life threatening disease.

## 1. Context

Prostate cancer is one of the most common cancers in male. However rates of detection of prostate cancers vary widely across the world, with Europe and the United States detecting higher frequency than South and East Asia. In China, the incidence rate is 1.6 cases per 100000, while 119.9 cases per 100000 in the USA (1). Prostate cancer tends to develop after the age of fifty in men, but unfortunately many patients do not have symptoms, they do not take treatment, and eventually die. The reasons behind this may be the slow growing cases of prostate cancer, and since older people may die of other causes such as heart/circulatory disease, pneumonia, other unconnected cancers, or old age. Although two-third cases of prostate cancers are slow growing, there are some cases of aggressive prostate cancers. Recent evidences from the Prostate, Lung, Colorectal, and Ovarian Cancer Screening Trial (PLCO), and the European Randomized Study of Screening for Prostate Cancer suggested a high rate of overdiagnosis, and overtreatment of prostate cancer, which causes a low mortality rate relative to the incidence rate over the last two decades (2, 3). Despite these results of the foregoing trials and the success, the high intervention rate of prostate cancer continues. Unfortunately, prevention may have little effect on disease-related mortality. Primarily, surgery, radiation therapy, and proton beam therapy are the current treatment options of prostate cancer. However, chemotherapy, hormonal therapy, cryosurgery, and high intensity focused ultrasound (HIFU) are also belonging to the treatment strategies, depending on clinical conditions, and outcomes. Also, the choice of treatment depends on the stages of the disease progression, the level of prostate specific antigen (PSA), the Gleason score among others. Patient's age, general health conditions, his interest about treatments, and their possible side-effects may also influence choosing among different treatment options. Any of the treatments may have significant side-effects, so the treatment discussions often focus on balancing the goals of therapy with the risks of lifestyle alterations. Dietary management, and other lifestyle modification of patients with prostate cancer have also shown some positive results to control, and prevent prostate cancer. Patients with prostate cancer are strongly recommended to work closely with their physicians, and use a combination of the treatment options when managing their prostate cancer (4, 5). The optimal management of prostate cancer still remains controversial. This review article summarizes the current treatment and prevention strategies with the protection of prostate cancer, which may be helpful to control and prevent this highly frequent life threatening disease.

## 2. Evidence Acquisition

### 2.1. Surgery

Surgery is not regarded as monotherapy in men with prostate cancer; rather it is a part of the multimodality approaches. Surgery is mainly suggested for high-risk locally advanced prostate carcinoma (6). Radical prostatectomy (7) and pelvic lymphadenectomy (PLDN) are mostly applicable surgery types in prostate cancer. Traditionally, RP for high-risk prostate cancer has been discouraged because of concerns regarding the side effects such as high rates of positive surgical margins, risk of lymph node metastasis, and high rates of PSA recurrence. However, surgery has been shown to be more beneficial than watchful waiting for mortality, risk of local progression, and risk of metastasis (8). Montie suggested that initial RP may have a role for treating high risk localized prostate cancer (9). After 8-10 years of following up, Bill-Axelson et al. (10) suggested that RP reduces disease-specific mortality, overall mortality, and the risks of metastasis, and local progression of prostate cancer. According to their study, the absolute reduction in the risk of death after 10 years was small, but the reductions in the risks of metastasis, and local tumor progression were substantial. Patients most likely to benefit from surgery include those with a biopsy Gleason score ≤ 8, the serum PSA level < 20 ng/ml, and the tumor ≤ cT3a (11); these criteria are currently recommended by the European Urology Association (5) for surgery in locally advanced prostate cancer (12). PLND is commonly suggested to perform during RP for high-risk prostate cancer (8) because 15% to 40% of nodes would have positive results (13). To detect the lymph node metastases in prostate cancer, PLND is the most reliable strategy, but its therapeutic benefit in prostate cancer management is still debatable (14). Zorn et al. (15) described PLND technique during robot-assisted RP in a cohort study to evaluate the nodal yield and perioperative outcomes, and they demonstrated the feasibility and low complication rate of robotic standard-template PLND with lymph node yields comparable to those with open PLND.

### 2.2. Radiation Therapy

After RP, radiotherapies are considered as the second major therapeutic modalities for localized high-risk prostate cancers. External-beam radiotherapy (EBRT), and brachytherapy are widely used treatment strategies for prostate cancer, which have a significant clinical and technological development in recent decades (16). Low-dose rate brachytherapy (LDRB) involves the permanent insertion of radioactive seeds with the half-life of 60 days under the ultrasound guidance (17). A small randomized trial comparing RP and LDRB for low-risk prostate cancer demonstrated equivalent outcomes, with a 5-year biochemical progression-free survival of 91.7% by brachytherapy versus 91.0% by surgery, however they produce different short-term sequelae of urinary disorders, and erective functions (18). Also these two strategies (brachytherapy and surgery) have similar cost profile for prostate cancer treatment in France (19). In high-dose rate brachytherapy (HDRB), there is a temporary insertion of applicators into the prostate to ensure a feeding of high energy source by different positions of prostate. This ensures a high dose of radiation to prostate gland with the minimized dose to bladder and bowel. HDRB can be used as monotherapy or in combinations with EBRT. Usually, HDRB offers a good treatment strategy for patients with more locally advanced disease (17). EBRT may be effective to every patient without distant metastases, and a life expectancy of at least 5-10 years (20). The advantage of a dose escalation up to the total doses of 76-78 Gy concerning biochemical tumor control has been showed by some randomized trials, which additionally concerns the disease-specific survival for high risk patients. Other randomized trials demonstrated the benefits of an additional adjuvant antiandrogen therapy to EBRT for patients with locally advanced cancers. A radiation dose of at least 74 Gy should be the standard of care for all men with localized prostate cancer who choose treatment with EBRT (16). However, the optimal dose of EBRT has not yet been established for these patients, and an argument can be made for additional dose escalation. For reducing metastases risk and increasing survival, Pinkawa (20) recommended an adjuvant postprostatectomy EBRT of the prostatic fossa with doses in the range of 60-66 Gy.

### 2.3. Proton Beam Therapy

Proton beam therapy (PBT) is one the types of EBRT which use ionizing radiation. The main advantage of proton beam therapy is its ability to localize the radiation dosage more precisely when compared to other types of radiation therapy. A particle accelerator is used to target the tumor with a beam of protons during the treating process. PBT allows an excellent dose distribution, with the additional benefit of no exit dose. These characteristics make PBT as an excellent choice for the treatment of prostate cancer (21). In a phase III trial by Shipley et al. (22), an increased dose with an external beam of 12.5% to 75.6% CGE (Cobalt Gray Equivalent) by a conformal proton boost compared to a conventional dose, significantly improved local control of cancer in patients with poorly differentiated prostate tumors. With an emphasis on the biochemical freedom from relapse, Slater et al. (23) analyzed the results of conformal proton radiation therapy for localized prostate cancer, and reported that conformal proton radiation therapy yields disease-free survival rates with a minimal rate of morbidity. Over the last ten years, proton beam therapy has increased the survival rate among patients with prostate cancer (24).

### 2.4. Cryosurgery

Cryosurgery is a treatment strategy where extreme cold is applied to destroy abnormal or diseased tissue, including prostate tumors. In this strategy, the supercooled liquid is sprayed on the diseased tissue by using liquid nitrogen as the cooling solution. For the treatment of localized low-risk prostate cancer, focal cryotherapy has emerged as a less morbid option, and obviously an interesting concept (25) Bahn et al.(26) retrospectively reviewed the efficacy and safety of the long-term experience with targeted cryoablation of prostate cancer (TCAP) in a series of 590 consecutive patients, who experienced TCAP as primary treatment for localized or locally advanced prostate cancer for 7 years at a community hospital. The outcome provided the compelling validation of TCAP as an effective treatment strategy for the locally confined, and locally advanced prostatic carcinoma (26). Hubosky et al. (27) critically examined patients at a single institution, who were receiving the third-generation cryosurgical treatment for localized prostate cancer. They reported that treatment success with cryosurgery varies with treatment outcome, morbidity profile, and quality of life parameters definition, but their results were comparable to other series in the regard of short-term cancer control. In that series of patients undergone third-generation cryosurgery, the complication rates were low; quality of life parameters of third-generation cryoablation were similar to second-generation series. Compared to brachytherapy, cryotherapy was found as less irritative, and obstructive voiding symptoms in the early post-treatment period, and it improved the urinary function after treatment (27). In a randomized, noninferiority trial to compare cryoablation with EBRT in patients with prostate cancer, after a long-term follow up the trend favored cryoablation/ cryosurgery (7).

### 2.5. Hormonal Therapy

Androgens are regarded as the fuel for hungry prostate tumor (28). Testosterone accounts for more than 90% of the systemic androgen function, and dihydrotestosterone (DHT) is its important variant (cytosolic) (29). The androgen receptor (AR) is a ligand-dependent transcription factor which acts in the nucleus of cells (30). The AR binds to testosterone and DHT with similar affinity, although DHT is a more potent androgen for structural and biochemical reasons (31). At normal concentrations, adrenal androgens have little effect on the prostate. Although activation of the AR by androgens is the most direct means of promoting prostatic growth, there are several surrogate pathways in prostate cancer. These pathways permit the AR to be activated, amplified, enhanced or bypassed without androgen stimulation, thus leading to the development of prostate cancer (32). Androgen deprivation therapy (ADT) with either medical or surgical approach is regarded as the initial treatment for metastatic prostate cancer. The beneficial clinical effects of ADT in men with symptomatic metastatic prostate cancer are rapid and dramatic (33). Huggins et al. (34) reported the dramatic clinical effects of suppressing serum testosterone levels in men with advanced prostate cancer. Inhibition of various hormones, receptors, or enzymes along the androgen production pathway is the basis of treatment. ADT is frequently used as the primary treatment for prostate cancer, particularly locally advanced and metastatic disease. It is also used as neoadjuvant, and adjuvant therapy, in combination with surgical or radiation therapy. ADT does not cure prostate cancer when used alone but is often the treatment modality of choice for palliative therapy. Although the emphasis of cancer treatment is typically focused on the cancer cells directly, an emerging concept in the treatment of prostate cancer is inhibition of prostatic stroma in addition to the tumor. The prostatic stroma has been shown to have a supportive role in prostate cancer, and may play a role in driving cells into a tumorigenic or invasive phenotype (29, 32). Initially, diethylstilbestrol was used for achieving androgen deprivation, but was replaced by luteinizing hormone-releasing hormone (LHRH) (33). Presently, medications used for ADT include estrogens, GnRH (Gonadotropin releasing hormone) agonist, GnRH antagonist, androgen receptor blockers, 5-alpha reductase inhibitors, adrenal androgen inhibitors and some others (29). Although ADT is very widely used, the role of ADT in the management of prostate cancer is highly controversial. Adverse events associated with LHRH agonists include the flare phenomenon, hot flashes, loss of libido, erectile dysfunction, depression, muscle wasting, anemia, and osteoporosis (33). Also, ADT reduced insulin sensitivity and increased body weight, serum cholesterol and triglyceride levels. A significant cardiac risk has also been shown, as neoadjuvant hormonal therapy given with radiation therapy has been shown to increase all-cause mortality in men with a history of coronary artery induced congestive heart failure or myocardial infarction; this effect was not seen in men with up to a single coronary artery disease risk factor (35). Fortunately, bilateral orchiectomy (an out-patient surgical procedure for the removal of the testicals) is associated with fewer side effects than medical ADT. Bilateral orchiectomy does increase the risk of diabetes, similar to GnRH agonists; however it does not appear to have the same increase in myocardial infarction, coronary heart disease, and cardiac death (36, 37).

### 2.6. Chemotherapy

Generally, chemotherapy is not regarded as the very effective way against prostate cancer. In fact, before mid-nineties of last century, it was thought that chemotherapy is not beneficial for prostate cancer. However, after that time, the use of chemotherapy in patients with hormone refractory prostate cancer (HRPC) has shown significant improvements in pain and quality of life, as well as decreases in PSA level (38). The common chemotherapeutic drugs used as the treatments of advanced prostate cancer include mitoxantrone, doxorubicin, vinblastine, paclitaxel, docetaxel, and some others. Mitoxantrone is an anthracenedione antineoplastic agent. Mitoxantrone plus prednisone (a pro drug) reduce pain and improve the quality of life in patients with advanced HRPC, but do not improve the survival rate. For metastatic HRPC, the combination of mitoxantrone, and prednisone is now approved as a second-line treatment. However this combination was regarded as the first line of treatment previously until the recent development of treatment strategy with the combination of docetaxel and prednisone, which has been shown to improve survival and disease-free period (39). A recent study confirmed that the survival rate of men with metastatic HRPC is significantly higher after the treatment with docetaxel and prednisone than that with mitoxantrone and prednisone (40). Docetaxelis is a clinically well-established antimitotic chemotherapeutic medication. This drug interferes with cell cycle by binding with the microtubules. It has also been found to influence the phosphorylation of oncoprotein bcl-2, blocks the apoptosis (41). The monotherapy with anthracyclines, doxorubicin or epirubicin, or their combination with other agents, have been used extensively in the treatment of HRPC, but the outcomes were controversial (42).

### 2.7. Dietary Strategies

Like many other disorders, the interactions between individual genetic susceptibility, and the life style background, including the diet, are responsible for cancer causation. Dietary modification is an important way to prevent cancer, because some dietary factors may contribute to a decrease in risk while others could cause an increase. Avoiding high fat and cholesterol may help to control or prevent prostate cancer, because dietary fat and cholesterol play an important role in the development of prostate cancer (43). Shirai et al. reported ω-6 polyunsaturated fatty acids to exert promotional effects in prostate carcinogenesis, and ω-3 polyunsaturated fatty acid-rich oils to suppress tumorigenesis (44). Freedland et al. reported that no-carbohydrate ketogenic diet could significantly reduce prostate cancer growth, and prolonged survival in xenograft model mice injected with LAPC-4 cells. This activity was associated with favorable changes in serum insulin and insulin-like growth factors (IGF) axis hormones relative to low-fat or Western diet (45). In laboratory studies, nutraceutical compounds most commonly show antioxidant properties combined with other antineoplastic actions. Because oxidative stress with androgen exposure and age-factor increase prostate cancer risk, dietary materials with antioxidants should be effective against prostate cancer (46-48). Reports from several studies reviewed by Shirai et al. (44), have suggested that isoflavones, carotenoids, and in particular lycopene, could be prostate cancer-preventive agents. However Peters et al. (49) suggested that lycopene is not effective for prostate cancer prevention. Dietary intake of selenium, which is present in a wide range of foods such as fish, meat, poultry, eggs, dairy products, grains, and some others, has been suggested to have a protective effect against prostate cancer (50). A Meta-analysis performed by Brinkman et al. suggested that men with low selenium levels are at increased risk of prostate cancer. Redman et al.(51) reported the inhibitory effect of selenomethionine on DU-145 prostate cancer cells through inducing apoptosis. However the effect of selenium on human trial is controversial. Meyer et al.(52) suggested that nutritional doses of antioxidant vitamins (like vitamin E), and minerals (like selenium) may help to the chemoprevention of prostate cancer. But, in clinical trials, vitamin E and selenium were not so effective. The Selenium and Vitamin E Cancer Prevention Trial (SELECT) was designed on 35,533 healthy men from American and African-American origin by Dunn et al.(53), and they observed that neither selenium nor vitamin E, alone or together, prevented prostate cancer in this heterogeneous population. However there are evidences that vitamin D improves the survival of patients with prostate cancer, and vitamin D appeared to be important in reducing the risk of prostate cancer over many years (54). A high vitamin B-6 intake has also been suggested to improve prostate cancer survival among men with a diagnosis of localized-stage disease (55). The American Dietetic Association and Dieticians of Canada reported a decreased incidence of prostate cancer for the vegetarians (56).

## 3. Discussion

As prostate cancer is one of the life threatening and most frequent case of disorders, proper treatment and other control strategies are of specific goals to many biomedical researchers. An integrated treatment strategy, which combines the local and systemic therapies, can be beneficial in the management of prostate cancer. However, the choice of treatment strategy is dependent on many factors, like patient preference, and quality of life aspects. It is expected that within a near future, the treatment approaches like surgery, radiation therapy, hormonal, and chemotherapy would be much more developed without minimal side effects. And most importantly, proper dietary management may keep away a person from prostate cancer risk.
